# Machine Learning for Digital Scoring of PRMT6 in Immunohistochemical Labeled Lung Cancer

**DOI:** 10.3390/cancers15184582

**Published:** 2023-09-15

**Authors:** Abeer M. Mahmoud, Eileen Brister, Odile David, Klara Valyi-Nagy, Maria Sverdlov, Peter H. Gann, Sage J. Kim

**Affiliations:** 1 Department of Medicine, Division of Endocrinology, College of Medicine, University of Illinois Cancer Center, University of Illinois Chicago, Chicago, IL 60612, USA; amahmo4@uic.edu; 2 Department of Kinesiology and Nutrition, College of Applied Health Sciences, University of Illinois Cancer Center, University of Illinois Chicago, Chicago, IL 60612, USA; 3 Department of Pathology, College of Medicine, University of Illinois Chicago, Chicago, IL 60612, USA; brister2@uic.edu (E.B.); odavid@uic.edu (O.D.); klaravn@uic.edu (K.V.-N.); pgann@uic.edu (P.H.G.); 4 Research Histology and Imaging Collaborative Core, College of Medicine, University of Illinois Chicago, Chicago, IL 60612, USA; mariasve@uic.edu; 5 Division of Health Policy & Administration, School of Public Health, University of Illinois Chicago, Chicago, IL 60612, USA

**Keywords:** lung cancer, immunohistochemistry, PRMT6, digital pathology, concordance

## Abstract

**Simple Summary:**

We trained and validated the machine learning digital scoring method of PRMT6 protein expression in lung cancer tissue samples. PRMT6 is an important biomarker for the progression of lung cancer; however, conventional pathologists’ manual scoring of its expression in large samples is time consuming, particularly when analyzing large sections of lung cancer tissue. Using HALO software, we optimized the digital method for scoring PRMT6 expression on immunohistochemically stained lung cancer tissue. Our optimized digital scoring showed excellent concordance with two pathologists using the immunoreactive scoring method. Our findings showed that digital scoring trained by pathologists can be a more efficient method with a high level of accuracy.

**Abstract:**

Lung cancer is the leading cause of cancer death in the U.S. Therefore, it is imperative to identify novel biomarkers for the early detection and progression of lung cancer. PRMT6 is associated with poor lung cancer prognosis. However, analyzing PRMT6 expression manually in large samples is time-consuming posing a significant limitation for processing this biomarker. To overcome this issue, we trained and validated an automated method for scoring PRMT6 in lung cancer tissues, which can then be used as the standard method in future larger cohorts to explore population-level associations between PRMT6 expression and sociodemographic/clinicopathologic characteristics. We evaluated the ability of a trained artificial intelligence (AI) algorithm to reproduce the PRMT6 immunoreactive scores obtained by pathologists. Our findings showed that tissue segmentation to cancer vs. non-cancer tissues was the most critical parameter, which required training and adjustment of the algorithm to prevent scoring non-cancer tissues or ignoring relevant cancer cells. The trained algorithm showed a high concordance with pathologists with a correlation coefficient of 0.88. The inter-rater agreement was significant, with an intraclass correlation of 0.95 and a scale reliability coefficient of 0.96. In conclusion, we successfully optimized a machine learning algorithm for scoring PRMT6 expression in lung cancer that matches the degree of accuracy of scoring by pathologists.

## 1. Introduction

The manual scoring of immunohistochemical (IHC) stained tissue sections by pathologists has traditionally been the gold standard for assessing protein/antigen expression. Nonetheless, several shortcomings have been identified with this method, such as the limited range of scoring, human errors, and lack of reproducibility. These limitations necessitate the development of automated approaches to analyze digital images, which may be more efficient and reproducible [1,2,3,4]. Digital scoring is expected to solve these problems since it can easily separate overlapping stains such as hematoxylin, which is widely used to counterstain nuclei, and 3,3′-diaminobenzidine (DAB), which is used to detect antigens [4]. This type of overlap frequently leads to an overestimated nuclear antigen expression. The digital scoring of IHC also aids in the detection and quantification of poorly expressed antigens, as well as subtle differences between samples that are difficult to detect with the naked eye yet have prognostic significance [5,6,7,8]. Aside from improving the accuracy of the scoring, digital platforms allow the predetermined algorithm parameters to be applied across cases, resulting in greater reproducibility and correlations with antigen expression levels. Finally, digital scoring is useful in managing large numbers of samples, such as tissue microarrays (TMAs), which would otherwise be time-consuming for pathologists to analyze manually. 

Despite these benefits, automated scoring is limited by the efficacy of the training algorithms. Digital scoring approaches are often highly impacted by tissue and staining artifacts, leading to inaccurate scoring when faced with uneven color patterns, overlapping tissue areas, fragmented nuclei, or complicated histological patterns [9].

Pathologists generally use an ordinal scale ranging from “0” to “3” to represent negative, weak, moderate, and strong staining, respectively. Some pathologists, however, have employed a more comprehensive scoring scale known as the “immunoreactive score (IRS)” to obtain semi-continuous scores. In the IRS approach, the intensity of staining is ordinarily graded from “0” to “3” and then multiplied by the percentage of positive cells reflecting that intensity level [10]. Using the IRS approach has been shown to enhance the concordance between manual and automated scoring in previous studies that examined hormone receptors in breast cancer tissues [11,12,13,14]. Similar results were observed in other malignancies, including colorectal, ovarian, prostate, and esophageal cancers [15,16,17]. 

Lung cancer has the highest mortality rate among cancers in the United States (U.S.), with approximately 228,000 new cases and 142,000 deaths per year, with non-small cell lung cancer (NSCLC) accounting for nearly 85% of lung cancer cases [18,19]. While advances in early detection and treatment have reduced lung cancer mortality, not all racial/ethnic groups benefited from the improvement [18,20,21,22], particularly in highly segregated urban cities such as Chicago [22,23,24]. Furthermore, the molecular mechanisms responsible for lung cancer’s initiation and promotion are not entirely understood. Therefore, there is a pressing need to identify novel biomarkers for the early detection and progression of lung cancer in order to develop more effective therapies for lung cancer. One mechanism that is frequently dysregulated in cancer is the post-translational modifications (PTM) of proteins. PTMs are known to impose functional diversity by fostering dynamic changes in protein structure and function, making them promising research and therapeutic prospects.

Protein methylation is catalyzed by PRMTs and is one of the most abundant post-translational modifications that occur in cellular proteins [25,26]. Ten mammalian PRMTs have been identified to date, of which PRMT1, CARM1, PRMT5, PRMT6, and PRMT9 are found to be highly expressed in several cancers and correlate with poor overall survival [27,28,29,30,31,32]. Among these markers, PRMT6 was shown to be related to lung cancer [33] and differentially expressed among racial/ethnic groups. Furthermore, in vivo mechanistic studies indicated that the lung-specific overexpression of PRMT6 in a murine model resulted in the spontaneous development of lung cancer and potentiated chemical carcinogen-induced lung cancer progression [34]. Previous clinical investigations reported PRMT6 overexpression in lung adenocarcinoma and other NSCLCs, which was associated with advanced clinical stage, lymph node metastasis, and poor survival rates [35,36]. The techniques of small molecule and peptide inhibitors that target PRMTs have evolved in recent years, emphasizing the clinical significance of investigating PRMTs, particularly in cancers with high mortality rates such as lung cancer. 

The purpose of this study is to validate automated PRMT6 scoring in lung cancer surgical specimens. Once validated, this automated scoring system could be used in larger cohorts to assess the association of PRMT6 expression in lung cancer with socioeconomic and clinicopathologic characteristics. To the best of our knowledge, there has not been a study exploring the manual-machine scoring concordance using the IRS approach in lung cancer biomarkers. In the current study, we used formalin-fixed, paraffin-embedded (FFPE) lung cancer tissue sections labeled with a specific antibody against PRMT6 to examine the ability of commercially available software algorithms (HALO software version 3.4 including the MiniNet AI and Nuclei Seg plugins for tissue and nuclear segmentation, respectively) to replicate the IRS obtained manually by two pathologists. 

## 2. Materials and Methods

### 2.1. Biological Samples

This study included 33 FFPE lung cancer tissue sections that were obtained retrospectively from the University of Illinois Tissue Bank. The parent study protocol was approved by the University of Illinois Chicago Institutional Review Board (IRB# 2021-1079). 

### 2.2. Immunohistochemical Staining

Serial sections from lung cancer tissue blocks (4 μm) were cut, deparaffinized in xylene twice, fpr 20 min each., and rehydrated by incubating them sequentially with 100%, 95%, 80%, and 60% ethanol followed by distilled water. Tissue sections were then subjected to the appropriate antigen retrieval (heating in a microwave for 10 min on medium settings) and non-specific binding blocking processes. This was followed by incubation with the PRMT6 primary (1:2000; rabbit polyclonal, catalog #A300-929; Biomol GmbH-Life Science Shop, Hamburg, Germany) for one hour at room temperature. The slides were then washed in Tris-based buffer (TBS) 1X three times and incubated with the appropriate anti-rabbit, HRP-conjugated secondary antibody for 30 min at room temperature. Finally, the sections were visualized with 3,3′-diaminobenzidine (DAB) and hematoxylin (counterstain). IHC staining was performed by the UIC Histology Core Facility and optimized by testing different sources and dilutions of the primary antibody and different methods of antigen retrieval.

### 2.3. Evaluation of Staining

After a consensus was reached about cut-off levels, two pathologists performed manual scoring of PRMT6 without knowledge of case outcomes. Immunoreactive scores (H-scores) were calculated as the sum of staining intensity (0, 1+, 2+, 3+) multiplied by the percentage of positive cells (0–100%) within each intensity category. The final scores ranged from 0 to 300. For digital analytics of the IHC stained sections, digital images (40× magnification (0.0625 μm^2^ per raw image pixel) were obtained using the Aperio AT2 scanner (Leica Biosystems, Deer Park, IL, USA) fitted with a 40×/0.75 Plan Apo objective lens (Olympus, Center Valley, PA, USA). Analysis was performed using HALO software version 3.4 (Indica Labs, Albuquerque, NM, USA). Samples were manually annotated to identify regions of interest and to exclude artifacts. Using a draw tool to outline and label regions as ground truth, an experienced non-pathologist researcher trained the MiniNet classifier in HALO to segment tissue into tumor, non-tumor, and background regions. The pretrained HALO AI Nuclei Segmenter network embedded in the Multiplex IHC module was used with minimal additional training to segment nuclei in all regions of interest. While training these networks, a visual review of segmentation accuracy, real-time cross- entropy readout, and validation metrics were used to determine segmentation error and the need for additional training examples. Multiplex IHC algorithms were then used to deconvolve hematoxylin and DAB and quantify DAB intensity in each cell.

Digital scoring was first performed on a set of 8 samples that the pathologist carefully selected to encompass both typical and atypical histologic patterns. Under the supervision of a pathologist (A.M.M), a non-pathologist AI researcher (E.B.) manually annotated tumor regions on whole-slide images utilizing Aperio’s annotation software (ImageScope, Aperio version 12.4.6). Image regions were annotated to represent three user-defined classes (carcinoma, stroma, and background) for automated tissue segmentation. These image regions served as input parameters for histologic pattern recognition training software to generate a training set. The effectiveness of tissue segmentation was enhanced by feedback to the AI researcher from one of the pathologists who manually scored the sections to improve the segmentation of cancer cells against stromal and immune cells. Based on this input, training regions were edited by the AI researcher to improve the AI classifier. For instance, in cases where the classifier algorithm erroneously identified carcinoma regions as stroma, the training was enhanced by using additional carcinoma annotation areas to more accurately depict the carcinoma class. This procedure of iteratively modifying annotations and re-executing the training algorithm was repeated until the classification reached its optimal state, which was visually confirmed by the pathologist. With feedback from the pathologists, thresholds were visually set to distinguish negative, weak, moderate, and strong stains. The adjusted thresholds based on the training set were then used across all samples. For each sample, the H-score was calculated as the sum of staining intensity (0, 1+, 2+, 3+) multiplied by the percentage of positive cells (0–100%) within each intensity category. When performance on the initial 8 samples reached a satisfactory level, the AI algorithm was applied to the full set of 33 samples.

### 2.4. Statistical Analysis 

To examine the inter-rater agreement, we used the intra-class correlation (ICC) among the 3 raters: two pathologists and the Multiplex IHC algorithm independently scored 33 cases. We used two-way random ICC because we have 3 consistent raters for 33 samples. This ICC assumes that the variance in the raters only adds noise to the estimate of the scores and that the average rater error would reach zero. Thus, while a particular rater might rate Case 1 high and Case 2 low, the rating should all even out across raters. We interpret the average rating for this analysis.

## 3. Results

### 3.1. Baseline Characteristics of the Study Subjects

The 33 samples consisted of 12 White, 20 Black, and 1 other races (Table 1). The mean age at the time of diagnosis was 64 years. In this sample, 21 cases were current smokers, 88 were former smokers, and 44 never smoked. Histological classifications included 24 adenocarcinoma, 88 squamous cell carcinoma, and 11 basaloid squamous cell carcinoma. 

### 3.2. PRMT6 Expression and Subcellular Localization in Lung Cancer Tissue

**Figure 1:**
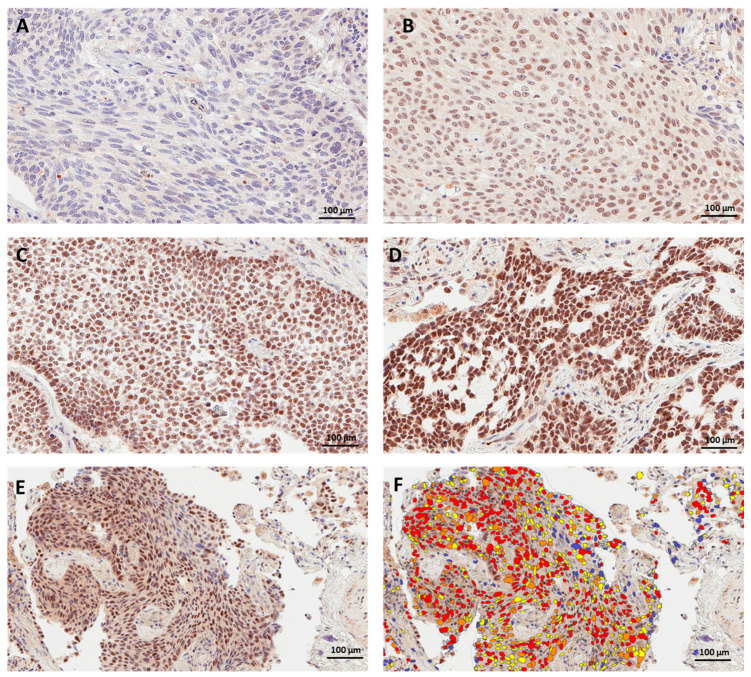
Immunohistochemical staining for PRMT6 in representative cases of (**A**) negative staining, (**B**) mild intensity, (**C**) moderate intensity, and (**D**) strong intensity. The conversion of various degrees of intensity in a representative section (**E**) into a heatmap for nuclear positivity (**F**), with blue representing negative staining, yellow representing mild positivity, orange representing moderate positivity, and red representing high positivity.

PRMT6 expression in immunohistochemically labeled lung cancer tissues was evaluated by two independent pathologists blinded to the demographic and clinical characteristics of corresponding patients. PRMT6 was found to be expressed predominantly in the nuclei of cancer cells, agreeing with previously reported localization in lung cancer [34]. A range of positivity was observed in stromal cells and infiltrating immune cells, but only the intensity of staining in tumor cells was assessed. The mean and median H-scores were 89 and 90 for Pathologist 1 and 78 and 84 for Pathologist 2. The agreement between the two pathologists was excellent, with ICC at 0.93. The average H-score corresponding to some demographic and clinicopathologic parameters in the current cohort is included in Table 1. We also grouped nuclear H-scores into above and below the mean H-score. Pathologist 1 scored 54.6% of 33 cases above the mean H-scores, while Pathologist 2 scored 48.5% of the cases above the mean H-score. Representative images of nuclear PRMT6 staining intensity among various lung cancer tissues, along with an example of digital scoring based on the staining intensity, are shown in Figure 1.

### 3.3. PRMT6 Expression in Lung Cancer Tissue Using Digital Scoring

**Figure 2:**
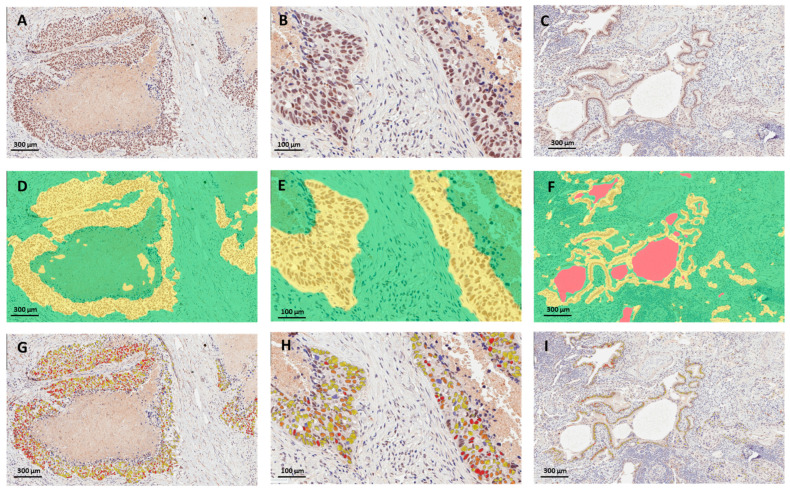
Image analysis workflow for IHC staining quantification. (**A**,**D**,**G**) are three different lung cancer tissue sections immunohistochemically labeled for PRMT6 (brown staining; DAB); blue hematoxylin staining labels the nuclei. (**B**,**E**,**H**) show corresponding tissue segmentation using HALO software version 3.4 that subclassified tumor areas into malignant epithelium (yellow), stroma (green), and empty space (pink). (**C**,**F**,**I**) show corresponding nuclear staining intensities pseudo-colored for negative (blue), mild (yellow), intermediate (orange), and strong (red) staining.

**Figure 3:**
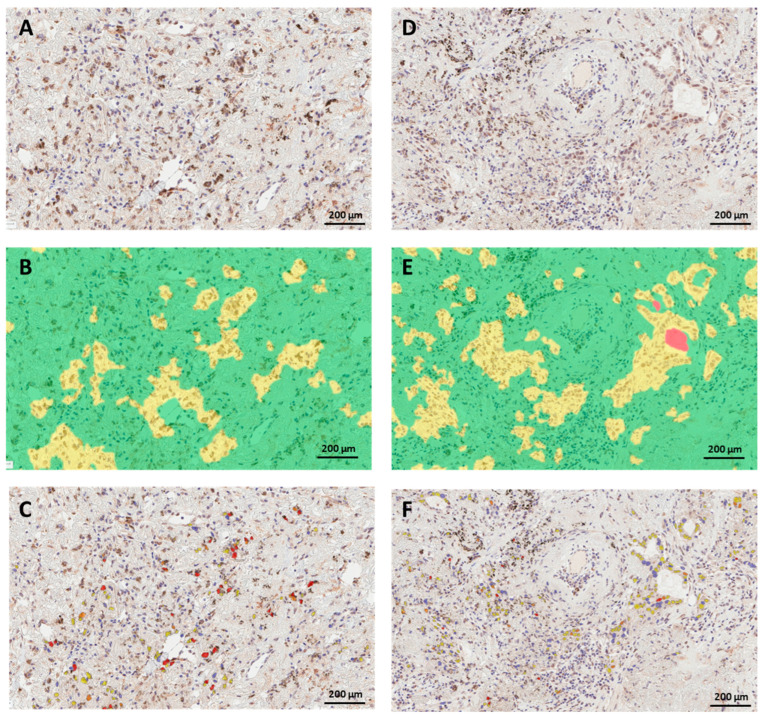
Challenging situations in digital image analysis of PRMT6 in lung cancer tissue. (**A**,**D**) are two different lung cancer tissue sections immunohistochemically labeled for PRMT6. (**B**,**E**) show corresponding tissue segmentation with incorrect annotation of areas of stromal cells as malignant epithelium (yellow). (**C**,**F**) show corresponding nuclear staining of erroneously annotated stromal cells. Color code: Green for the stroma, yellow for cancer cells, red for the background.

**Figure 4:**
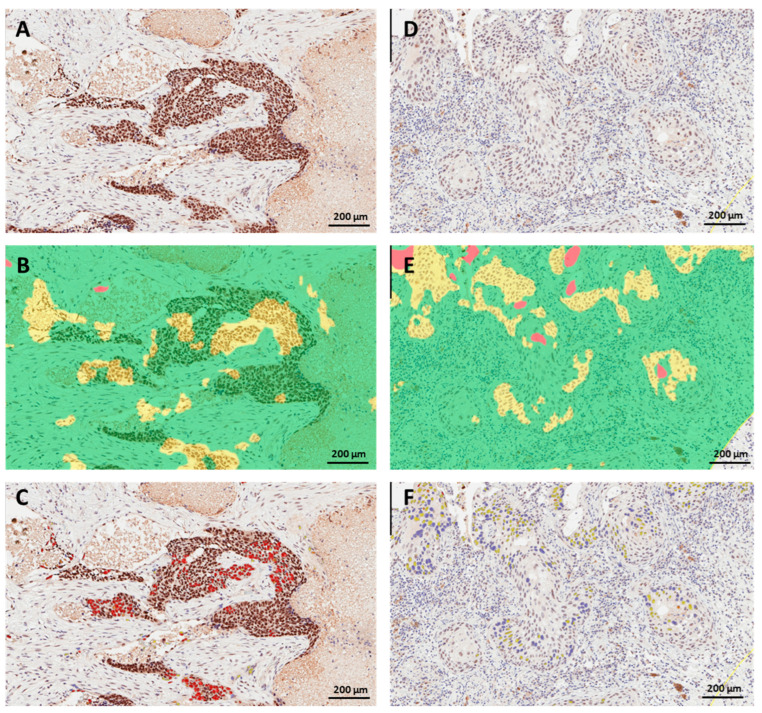
Challenging situations in digital image analysis of PRMT6 in lung cancer tissue. (**A**,**D**) are two different lung cancer tissue sections immunohistochemically labeled for PRMT6; malignant cells were manually annotated. (**B**,**E**) show corresponding tissue segmentation with the incorrect exclusion of areas of malignant epithelium that are spindle-shaped. (**C**,**F**) show a lack of nuclear scoring of the excluded malignant epithelial cells. Color code: Green for the stroma, yellow for cancer cells, red for the background.

The mean digital H-score was 94 and the median was 85 (Table 1). When we dichotomized nuclear H-scores at the sample mean, 45.5% of lung cancer cases had high PRMT6 nuclear staining. The continuous and dichotomized digital H-scores corresponding to some demographic and clinicopathologic parameters in the current cohort are included in Table 1. Representative images of the procedure for digital image analysis (tissue segmentation followed by nuclear color intensity analysis) of three examples are presented in Figure 2. The images in Figure 2 depict the ideal circumstances, when flawless tissue segmentation was performed, followed by nuclear scoring. The issues that arise with digital scoring are mostly associated with tissue segmentation and the distinction of tumor cells and non-tumor cells, such as stromal cells, immune cells, and artifacts. Figure 3 depicts two instances in which stromal cells and immune cells were picked erroneously during tissue segmentation. Depending on the level of expression of the biomarker in non-tumor cells, this might lead to either overestimated or underestimated scores of PRMT6 in cancer cells. The second example is that some portions of the tumor tissues are not annotated as malignant epithelial cells because they have certain cell characteristics and a resemblance to stomal cells that were excluded during the training of the algorithm, as shown in Figure 4.

### 3.4. Concordance between Manual and Digital Scoring of Nuclear PRMT6 Expression in Lung Cancer Tissues

Lung cancer sections were analyzed by two different observers via the digital imaging software, HALO version 3.4. For each scoring system, descriptive and variation analyses were performed (Table 2). 

To verify concordance and calculate the correlation, the scores obtained from the three evaluators were plotted. The correlation between the two pathologists was strong with a correlation coefficient (*r*) of 0.93. A high correlation was also observed between the pathologists’ scores and the digital imaging scores with a correlation coefficient (*r*) of 0.88. 

An excellent inter-rater agreement among the three scoring methods was observed, with ICC at 0.95, and the scale reliability coefficient is 0.96. 

We then applied the Bland–Altman analysis. Pathologists 1 and 2 showed a mean difference of 8.2 (SD: 19.8, 95% CI, 0.9581, 15.4935), Pathologist 1 and the digital system showed a mean difference of −5.2 (SD: 25.4, 95% CI, −14.2146, 3.8009), and finally Pathologist 2 and the digital scoring system showed a mean difference of −14.4 (SD: 25.4, 95% CI, −24.3636, −4.5096). The scores demonstrated excellent agreement between the two pathologists and with the digital analysis system. 

Scores were then dichotomized into “high PRMT6” and “low PRMT6” based on the average score in each scoring system. High PRMT6 was identified in 18/33, 16/33, and 15/33 cases by Pathologist 1, Pathologist 2, and the AI-based score, respectively. Cohen’s κ was performed to determine if there was an agreement between the three scoring systems regarding the classification of tumor tissues to PRMT6 high vs. PRMT6 low. There was a strong agreement between Pathologist 1 and Pathologist 2 (κ = 0.82, *p* < 0.001) and the digital score (κ = 0.82, *p* < 0.001), and a moderate agreement between Pathologist 2 and the digital score (κ = 0.64, *p* < 0.001). 

## 4. Discussion 

The findings of this study demonstrated that training algorithms for AI-based scoring can successfully replicate the level of accuracy of pathologists’ manual scoring of PRMT6 expression in lung cancer tissues. AI-based approaches to IHC scoring can be a reliable and efficient way to deal with a large amount of tissue samples. Digital scoring using HALO software version 3.4 (supported by the MiniNet AI and Nuclei Seg modules for tissue and nuclear segmentation, respectively) showed excellent concordance with two pathologists using the immunoreactive score method. However, we showed that the key to training machines improving their accuracy is the pathologist’s input and adjustment to the initial AI-based scoring. One important adjustment was in the tissue segmentation to cancer tissues versus non-cancer tissues to avoid errors coming from scoring non-cancer tissues or missing cancer cells. 

We saw that one of our pathologists tended to score “weak (1)” nuclear staining as “negative (0)”, resulting in a slightly lower score mean, compared with the other pathologist and the digital score. Rimm et al. [3] provided an earlier example of this issue when they demonstrated the bimodal distribution of pathologists’ visual score as a result of overcalling very weak staining as “negative”. Due to issues such as these, research may shift toward genuinely quantitative digital methods capable of detecting weak staining, particularly in the presence of the hematoxylin nuclear staining typically used in IHC methods. These methods would be particularly helpful in situations when the protein of interest is not abundantly produced.

It is important to note that digital scoring has its drawbacks, particularly concerning IHC staining. Digital scoring may be affected by artifacts such as nonspecific signals or altered tissue morphology when tissues are inadequately preserved or if antigenic retrieval or antibody dilution is suboptimal. The presence of air bubbles during the mounting process of the slide is another potential source of false-negative results. Examining how the digital system handles such situations is crucial. The software should be able to spot artifacts and avoid them, but this is not always the case. Manually excluding the problematic regions is always an option in these circumstances, although doing so would reduce the analysis throughout. Therefore, to attain the highest level of data precision, it is essential to conduct post-process evaluations at each stage for at least the initial few samples, until machine learning is optimized. 

Another potential source of challenge could be tissue segmentation, particularly in tumors characterized by heterogeneity in cell shape, nuclear size, and morphology, or tumors with multiple histological subtypes exhibiting distinct patterns. In the current study, the algorithm erroneously included non-tumoral regions such as stromal or immune cells (Figure 3) or excluded tumoral regions (Figure 4), when the tumor had a basaloid nature (spindle-shaped cells with hyperchromatic nuclei and little cytoplasm). Adjusting the parameters of the algorithms used to detect the tissue of interest or manually excluding unwanted regions could solve these issues. While these modifications may require pathologist oversight, they are still more efficient than having a pathologist manually score the entire section. In the current investigation, the supervision of digital image analysis by a pathologist required significantly less time than the manual scoring of tissue sections.

Bulk readings of lung tissue sections for PRMT6 protein expression may be required to advance social epigenetic research linking social exposure and biophysical consequences. Visual scoring by pathologists has traditionally been used, but with research involving large numbers of tissue samples, the availability and efficiency of pathologists can be severely limited. Accordingly, this study showed an alternative approach to PRMT6 scoring in lung cancer surgical specimens, which is automated and reliable. Along with rapid advancements in machine learning technology, our approach has the potential to become the gold standard for large-scale research assessing the relationships between social determinants of health and clinicopathologic outcomes. One of the limitations of the present investigation pertains to the limited sample size. Nevertheless, the use of entire lung cancer sections facilitated the examination of bigger sections of tumor tissues that were populated by a substantial number of cancer cells, in contrast to the tissue microarrays that are typically used in machine learning studies. Other limitations of this study include the use of tumors with a fragmented or irregular growth pattern, which could influence the pattern or intensity of PRMT6 staining. Additionally, in lung cancer, intra- and inter-tumoral heterogeneity in cell morphology and pattern is common and constituted an additional challenge [37]. This heterogeneity was particularly evident in our sample set among various histological subtypes, some of which were instrumental in optimizing the algorithm while others, particularly those with a basaloid nature, were challenging to distinguish from stromal cells. It is important to emphasize that the AI’s scoring capability is constrained by the training parameters established by the researchers. These parameters include the staining intensity threshold, which is influenced by factors such as the type and dilution of the antibody used, as well as the staining methods and conditions. Consequently, additional training will be necessary if modifications to the antibody or staining conditions are anticipated. 

PRMT6 is a crucial biomarker for the progression of lung cancer, and recently developed small molecule and peptide inhibitors of PRMTs highlight the therapeutic significance of studying PRMTs, especially in high-mortality diseases such as lung cancer. Moreover, given its connection to stress hormonal pathways, PRMT6 is an attractive candidate for studying the biological mechanisms underlying racial/ethnic disparities in lung cancer development and prognosis. Nevertheless, utilizing population health principles to biological basic science techniques introduces a challenge, especially with a large number of cases. This would be particularly difficult in the case of malignancies with molecular heterogeneity, such as lung cancer. The primary motivation of this study was to explore ways to address this limitation of manual scoring with the digital scoring of PERMT6 in lung cancer, which could enable larger biomarker assessment studies.

## 5. Conclusions and Future Perspectives

To the best of our knowledge, this is the first study to examine the concordance between the manual and digital assessment of PRMT6 in lung cancer tissue. Our data revealed a high level of concordance between the automated scoring system and the manual scoring performed by two pathologists independently. Automated IHC analysis is an effective alternative to manual scoring and more accurate and consistent in cases of less abundant biomarkers. However, quality control steps are required to exclude artifact-containing regions and non-relevant regions. 

There are two components to the inaccuracies of machine classification: the quality and accuracy of the data input to AI algorithms and the ability of AI programs to turn those annotations into accurate pattern recognition. In order for the AI algorithms to perform well, pathologists need to be involved in the training phase of machine learning. Improving AI’s ability to accurately classify less clear and less typical regions would be essential. Nonetheless, AI-based scoring can be an effective and scalable approach for larger-scale studies.

The objective of this study was to verify the accuracy of automated PRMT6 scoring in lung cancer surgical specimens, with the intention of developing it as the standardized approach for evaluating PRMT6 expression in lung cancer. This standardized method can then be applied in larger cohorts to investigate the correlation between PRMT6 expression in lung cancer and socioeconomic factors, as well as clinicopathologic characteristics. Furthermore, the present platform will facilitate protein-level multigene expression studies by leveraging the existing multiplex IHC techniques, which involve immunofluorescence, destaining and restaining methods, and the use of numerous chromogens. This type of research is expected to facilitate the evaluation of the concurrent expression of PRMT6 and associated biomarkers. 

## Figures and Tables

**Table 1:** PRMT6 score among different demographic and tumor-related characteristics.

	N	Mean Score			N (%) of High PRMT6 Cases ^a^		
		Pathologist 1	Pathologist 2	AI-Based	Pathologist 1	Pathologist 2	AI-Based
**Total**	33	89	78	94	18 (54.6)	16 (48.5)	15 (45.5)
**Self-reported race/ethnicity**							
Blacks	20	101	90	101	15 (75.0)	12 (60.0)	12 (60.0)
Whites	12	76	65	86	3 (25.0)	4 (33.3)	3 (25.0)
Other	1	10	25	52	0 (0.0)	0 (0.0)	0 (0.0)
**Age at diagnosis**							
≤64 years	19	88	75	96	9 (47.4)	8 (42.1)	8 (42.1)
>65 years	14	90	82	92	9 (64.3)	8 (57.1)	7 (50.0)
**Smoking status**							
Current smokers	21	94	80	98	13 (61.9)	12 (57.1)	11 (52.4)
Former smokers	8	92	79	95	5 (62.5)	4 (50.0)	4 (50.0)
Never smokers	4	59	68	75	0 (0.0)	0 (0.0)	0 (0.0)
**Histological subtypes**							
Squamous cell carcinoma	8	73		70	3 (37.5)	3 (37.5)	2 (25.0)
Basaloid squamous cell carcinoma	1	165	145	201	1 (100.0)	1 (100.0)	1 (100.0)
Adenocarcinoma	24	91		98	14 (58.3)	12 (50.0)	12 (50.0)

^a^ High PRMT6 includes cases scored above the average H-score.

**Table 2:** Descriptive and variation analyses of the three scoring systems.

	Pathologist 1	Pathologist 2	AI-Based
N	33	31	33
Mean	89.1	78.2	94.3
Standard Deviation	53.5	54.9	41.7
Range	10.0–200.0	0–180.0	19.7–200.8
Coefficient of Variation, %	60.0	70.2	44.2

## Data Availability

The article contains all of the findings obtained from the study.
